# Editorial: Thyroid function and its interaction with metabolic molecules

**DOI:** 10.3389/fendo.2023.1249218

**Published:** 2023-08-09

**Authors:** Miklós Bodor, Emese Mezősi

**Affiliations:** ^1^ Division of Endocrinology, Department of Medicine, Faculty of Medicine, University of Debrecen, Debrecen, Hungary; ^2^ Department of Clinical Basics, Faculty of Pharmacy, University of Debrecen, Debrecen, Hungary; ^3^ 1^st^ Department of Internal Medicine, Clinical Center, Medical School, University of Pécs, Pécs, Hungary

**Keywords:** thyroid, metabolic molecules, hypothyroidism, lipid, goiter, Hashimoto autoimmune thyroiditis

The hormone production of the thyroid gland affects the function of all other tissues and the body homeostasis in general. It has an enormous impact on lipid metabolism, development of atherosclerosis, body weight and also affects other hormone productions (1). Thus, the lately performed significant amount of research focusing on the interactions between the thyroid gland and metabolic factors revealed several important connections and led to a better understanding of the underlying mechanisms and led to novel treatment methods (2, 3).

This Research Topic offers new insights in the complex connection between the thyroid gland and metabolic factors.

It is well known that thyroid dysfunction and alterations in the lipid homeostasis are of great importance in the development of cardiovascular morbidities. Han et al. studied the impact of menopause on the connection between the thyroid and lipid parameters on data obtained from an impressive number of more than eighty thousand individuals. They found that hypothyroidism increased the risk of development of hypertriglyceridemia in both premenopausal and postmenopausal women, moreover, they showed that subclinical hypothyroidism has a negative impact on triglyceride and low-density lipoprotein cholesterol levels, and in contrary, subclinical hyperthyroidism does not affect the blood lipid values.

The benign diffuse goiter is a common finding among thyroid patients and the general population, however, the pathophysiology and the exact mechanism that leads to the development of goiter still remains to be elucidated. Benabdelkamel et al. (4) found that patients with simple goiter present a different protein expression pattern with significant reduction in thyroglobin production and elevation in enzymes involved in enhancing the redox state and thus regulating the oxidative balance of the thyrocytes.

Recently several studies were published regarding the pro-atherogenic effect of thyroid dysfunction, causing derangements in the endothelial cell function. Yao et al. investigated the expression patterns of serum MicroRNAs in patients with subclinical hypothyroidism and found that miR-21-5p may be involved in the process of development of atherosclerosis and thus may be a sensitive marker for the risk assessment of patients with atherosclerotic disease.

Fluid-electrolyte imbalances, including impaired free water excretion, are common in patients with hypothyroidism. The paper by Gergics et al. investigated the interactions of thyroid function, apelin and copeptin in the ion homeostasis in patients with hypothyroidism and found a marked decrease of serum apelin level in transitory severe hypothyroidism while the copeptin level remained unchanged. Also, they showed that copeptin and TSH are independent predictors of apelin concentration.

The regulation of thyroid hormone effects on tissue level is much more complex than is reflected by the serum levels of TSH and thyroid hormones. In the review of Jing and Zhang, a synthesis of the underlying mechanisms affecting thyroid hormone homeostasis is found. They describe the intrathyroidal feedforward and feedback network regulation explaining the Wolff-Chaikoff and Plummer effects and thyroglobulin-mediated regulation. Their work provides useful tools to better understand the pathophysiological processes, therapy response, and environmental disruptors’ effects.

The metabolic network has a fundamental role in many pathophysiological processes and abnormal metabolites contribute to the progression of autoimmune disorders. Jiang et al. were the first to analyze the serum metabolites in different clinical stages of Hashimoto’s thyroiditis (HT) and published in this Research Topic of Frontiers in Endocrinology. The 219 metabolites were assayed, and significant differences were found in 21 metabolites. Fatty acid degradation, arginine, and proline metabolism differed between euthyroid HT patients and the control group. Valine, leucine, and isoleucine metabolism were abnormal in subclinical hypothyroid patients compared to euthyroid ones. They concluded that the metabolic network is abnormal in the early stage of HT and further alterations are detected during the development of hypothyroidism. It is an open question whether these changes are only the markers of the disorder or active participants in the development of HT and progression to hypothyroidism.

HT is the most common organ-specific autoimmune disorder; the constantly increasing incidence of HT with age is a matter of speculation that accumulating impairs may contribute to the defect of the immune system. The relationship of HT with obesity, metabolic syndrome, type 2 diabetes, and PCOS was also intensively studied. Advanced glycation end products (AGEs) are the result of non-enzymatic glycation and oxidation of proteins, nucleic acids, and lipids, they have a definitive role in aging and the development of complications in glucometabolic disorders. They were first investigated in relation to type 2 diabetes but their role in disrupting physiologic processes is probably much more general. The possible involvement of AGEs in the development and progression of HT is new concept and literature data are sparse. The paper of Csiha et al. investigated the association of serum AGE and the soluble receptor for AGE (sRAGE) with thyroid function in patients with HT on levothyroxine replacement. They have found that the mean AGE level was lower and the sRAGE concentration was higher in the serum of HT patients compared to healthy controls. This favorable AGE/sRAGE balance correlated with the lower TSH and higher fT3 levels within the reference range. A previous study detected higher AGE and lower sRAGE levels in newly diagnosed euthyroid HT patients (Ruggeri RM). The authors assumed that levothyroxine replacement may be responsible for the more favorable AGE/sRAGE pattern. This question should be further investigated in a self-controlled study before and after levothyroxine treatment but if these results are confirmed, it may be an important argument supporting the early treatment of subclinical hypothyroidism due to HT.

## Author contributions

All authors listed have made a substantial, direct, and intellectual contribution to the work and approved it for publication.
